# KEEPING THE FAITH IN ADVERSITY: UNPACKING THE PARADOX OF INTENSIVE CAREGIVING

**DOI:** 10.1093/geroni/igae098.0643

**Published:** 2024-12-31

**Authors:** Ad Maulod, Nur ‘Atiqah Mohd Farhan, Sasha Rouse, Rahul Malhotra

**Affiliations:** Duke-NUS Medical School, Singapore, Singapore; Duke-NUS Medical School, Singapore, Singapore; Duke-NUS Medical School, Singapore, Singapore; Duke-NUS Medical School, Singapore, Singapore

## Abstract

Striving to live an authentic life while fulfilling caregiving duties is a common challenge among family caregivers. Previously, based on the distribution of the burden and benefits of caregiving, we identified four distinct archetypes – balanced (40%), satisfied (33%), intensive (17%) and dissatisfied (10%) – among family caregivers of older adults in Singapore. “Intensive” caregivers were most burdened in terms of poor health and financial strain yet continued to express caregiving as a highly rewarding endeavor, reporting benefits which were similar or greater than the other archetypes. In this paper, we interrogate this paradox by exploring the experiences of “intensive” caregivers through a one-year longitudinal qualitative study (Quali-T, N=39) conducted with caregivers across the different archetypes. Study findings reveal complex narratives of “intensive” caregivers seeking rewards to justify their burden. A karmic belief in intangible spiritual benefits seems to be the key explanation for this phenomenon. While the supportive effects of spirituality in overcoming caregiving challenges have been studied at length, we identify the intricate processes that occur when highly stressed caregivers negotiate meaning out of their circumstances, in relation to their personal need for spiritual well-being and authenticity. While spiritual support can be profoundly comforting for many caregivers, it is not a panacea to address all the challenges they face. By addressing the spiritual dimension of care in parallel to other aspects of caregiver support, healthcare and social service providers can offer more comprehensive person-centered care that supports caregivers’ holistically.

